# Peroral submucosal endoscopic stricturotomy – a novel third-space approach for a complex anastomotic stricture

**DOI:** 10.1055/a-2063-3465

**Published:** 2023-04-21

**Authors:** Alanna Ebigbo, Sandra Nagl, Markus W. Scheppach, Helmut Messmann

**Affiliations:** Department of Gastroenterology, University Hospital Augsburg, Augsburg, Germany

There are various options for the endoscopic treatment of benign anastomotic strictures of the esophagus, including balloon dilation or self-expanding metal stents. For complex refractory strictures, endoscopic radial incision (ERI) has been proposed 1 .

In our video case, we demonstrate the third-space endoscopic treatment of a complex, angulated, and subtotal anastomotic stricture in a 67-year-old patient following neoadjuvant radiochemotherapy and total esophagectomy due to squamous cell carcinoma (SCC, pT1b). Recurrent balloon dilations had been unsuccessful and placement of a fully covered metal stent did not retain luminal restoration. He was referred to our unit for further treatment, but because of the complex nature of the stricture, ERI was deemed not feasible. A third-space approach, per-oral submucosal endoscopic stricturotomy, to enhance stricturotomy was performed ( Video 1 ).

**Video 1** Peroral submucosal endoscopic stricturotomy for endoscopic treatment of a complex anastomotic stricture.


First, a short submucosal tunnel was dissected adjacent to the stricture. After tunnel dissection, a complete stricturotomy, including the mucosa and the fibromuscular bridge, was done. Finally, a wide opening of the anastomotic stricture with endoscopic passage towards the gastric pull-up was achieved. Initial tunneling made the depth and extent of stricturotomy more visible. Third-space endoscopic techniques have been successfully implemented to treat endoluminal obstruction, including peroral endoscopic tunneling for restoration of the esophagus (POETRE) and endoscopic tunneled stricturotomy 2 3 . Peroral submucosal endoscopic stricturotomy, as proposed here, may be a safe and effective approach for treating subtotal anastomotic strictures of the esophagus.

Endoscopy_UCTN_Code_TTT_1AO_2AN

## Notes

Endoscopy E-Videos

https://eref.thieme.de/e-videos
*E-Videos* is an open access online section of the journal *Endoscopy* , reporting on interesting cases and new techniques in gastroenterological endoscopy. All papers include a high-quality video and are published with a Creative Commons CC-BY license. Endoscopy E-Videos qualify for HINARI discounts and waivers and eligibility is automatically checked during the submission process. We grant 100% waivers to articles whose corresponding authors are based in Group A countries and 50% waivers to those who are based in Group B countries as classified by Research4Life (see: https://www.research4life.org/access/eligibility/ ). This section has its own submission website at https://mc.manuscriptcentral.com/e-videos
